# Electrophysiological Evidence for Domain-General Processes in Task-Switching

**DOI:** 10.3389/fnhum.2016.00124

**Published:** 2016-03-21

**Authors:** Mariagrazia Capizzi, Ettore Ambrosini, Sandra Arbula, Ilaria Mazzonetto, Antonino Vallesi

**Affiliations:** ^1^Department of Neuroscience, Universitá degli Studi di PadovaPadova, Italy; ^2^Department of General Psychology, Universitá degli Studi di PadovaPadova, Italy; ^3^Department of Information Engineering, Universitá degli Studi di PadovaPadova, Italy; ^4^Centro Neuroscienze Cognitive, Universitá degli Studi di PadovaPadova, Italy

**Keywords:** executive functions, spatial domain, verbal domain, source reconstruction, event-related potentials, prefrontal cortex

## Abstract

The ability to flexibly switch between tasks is a hallmark of cognitive control. Despite previous studies that have investigated whether different task-switching types would be mediated by distinct or overlapping neural mechanisms, no definitive consensus has been reached on this question yet. Here, we aimed at directly addressing this issue by recording the event-related potentials (ERPs) elicited by two types of task-switching occurring in the context of spatial and verbal cognitive domains. Source analysis was also applied to the ERP data in order to track the spatial dynamics of brain activity underlying task-switching abilities. In separate blocks of trials, participants had to perform either spatial or verbal switching tasks both of which employed the same type of stimuli. The ERP analysis, which was carried out through a channel- and time-uninformed mass univariate approach, showed no significant differences between the spatial and verbal domains in the modulation of switch and repeat trials. Specifically, relative to repeat trials, switch trials in both domains were associated with a first larger positivity developing over left parieto-occipital electrodes and with a subsequent larger negativity distributed over mid-left fronto-central sites. The source analysis reconstruction for the two ERP components complemented these findings by highlighting the involvement of left-lateralized prefrontal areas in task-switching. Overall, our results join and extend recent research confirming the existence of left-lateralized domain-general task-switching processes.

## Introduction

It is well-established that switching between two or more tasks comes at a great price in terms of accuracy and response speed. In a typical task-switching paradigm, participants have to repeat the same task or switch to a different one on the basis of a fixed order (i.e., the alternating runs paradigm; e.g., Rogers and Monsell, 1995) or according to an instructional cue that can be presented either in advance of or simultaneously with the target (i.e., the cued task-switching paradigm; e.g., Meiran, 1996). A robust finding that emerges in these kinds of experimental manipulations is the “switch cost”, which refers to a performance decrement in response time (RT) and accuracy on switch trials compared to repeat ones (see Monsell, 2003; Kiesel et al., 2010; Vandierendonck et al., 2010, for reviews).

Different theoretical formulations have been put forward to account for the switch cost. Amongst these, a popular hypothesis holds that switch trials are more demanding and time-consuming than repeat trials because they require the suppression of the task-set that was active on the preceding trial and the reconfiguration of a new relevant task-set (e.g., Rogers and Monsell, 1995; but see Allport et al., 1994; Wylie and Allport, 2000, for a different view). Under this task-set reconfiguration theory, task-switching represents a useful tool to investigate high-level cognitive operations. Several functional magnetic resonance imaging (fMRI) studies have indeed localized the ability to flexibly switch between tasks to networks controlling executive functions, such as fronto-parietal regions (e.g., Dove et al., 2000; Brass and von Cramon, 2004; Kim et al., 2011; Philipp et al., 2013). Converging evidence not only from neuroimaging but also from neuropsychological studies especially points to the importance of left-lateralized fronto-parietal areas (e.g., Aron et al., 2004; Shallice et al., 2008). A recent meta-analysis of neuroimaging studies by Kim et al. (2012) further supports the hypothesis that the left hemisphere is dominant for task-switching independently of the type of switch to be performed. The authors explored three types of task-switching (perceptual, response, and context switching) and found that the left inferior frontal junction (IFJ) and posterior parietal cortex (PPC) were the only regions commonly activated by the three task-switching tasks. Conversely, other regions within the fronto-parietal network were preferentially associated with the three types of tasks. These results thus suggest that both domain-general and domain-dependent neural mechanisms can contribute to task-switching.

Direct support for the specialization of the left hemisphere in task-switching also comes from a recent fMRI study by our group in which participants had to perform either a spatial or a verbal switching task on the same class of stimuli (Vallesi et al., 2015). A conjunction analysis showed that along with bilateral supplementary motor area, task-switching in both spatial and verbal cognitive domains activated left fronto-parietal regions regardless of the right and left hemispheric lateralization patterns usually associated with spatial and verbal processing, respectively (e.g., Boulinguez et al., 2003; Corballis, 2009; Corbetta and Shulman, 2011; Fairhall and Caramazza, 2013). Hence, this study also speaks in favor of a domain-general task-switching mechanism above and beyond specific domain-dependent ones. However, since Vallesi et al. (2015) compared blocks of single, all-repeated trials and mixed blocks of alternated trials (block design), it is difficult to generalize this conclusion to the transient activity elicited by switch compared to repeat trials. Moreover, it should be considered that the poor temporal resolution of fMRI does not tell us much about the temporal dynamics underlying task-switching mechanisms and whether they may change substantially across domains. Thus, exploring the electrophysiological correlates of spatial and verbal task-switching could add further information to that gathered from a block fMRI approach on the domain-general and/or domain-dependent nature of this key executive function (see also Muhle-Karbe et al., 2014).

To this end, in the current study we used the same experimental paradigm as in Vallesi et al. (2015) but exploited the excellent temporal resolution of event-related potentials (ERPs) to directly compare the time course and the modulatory effects associated with switch and repeat trials in the context of the spatial and verbal domains. Distributed source analysis was also applied to the ERP data to reconstruct the putative cortical generators of the ERP components of interest. Combining ERPs and source localization procedures allowed us to depict both the temporal and spatial dynamics of brain activity underlying task-switching abilities.

Several ERP studies of cued task-switching have shown that the neural activity associated with switch and repeat trials can be differentiated during the time interval that separates cue and target presentation. For instance, cues signaling a switch tend to elicit a larger posterior-parietal positivity as compared to cues indicating a task repetition (hereafter, “switch positivity”; see Karayanidis et al., 2010, and Karayanidis and Jamadar, 2014, for reviews; Kieffaber and Hetrick, 2005; Miniussi et al., 2005; Nicholson et al., 2005; Li et al., 2012; Kopp et al., 2014). Moreover, after target onset switch trials are typically associated with a larger fronto-central N2 and a smaller centro-parietal P3 than repeat trials (e.g., Kieffaber and Hetrick, 2005; Nicholson et al., 2005; Lavric et al., 2008; Periáñez and Barceló, 2009; Gajewski and Falkenstein, 2011; Hsieh and Wu, 2011). The cue-locked switch positivity has been theoretically interpreted within the task-set reconfiguration theory as an index of a “switch-specific reconfiguration process” (Karayanidis et al., 2011). That is, a switch in task would firstly require reconfiguring the task-set that was active in the previous trial and this would be reflected by a relative increase in the cue-locked switch positivity amplitude. In support of the task-set reconfiguration view, it has been reported that the switch positivity is postponed until target onset when task-set reconfiguration cannot be completed beforehand, as is case with a short or absent cue-target interval (e.g., Nicholson et al., 2005, 2006; Friedman et al., 2008; Karayanidis et al., 2009). In both cases, in fact, reconfiguration is initiated only after the presentation of the target, whose characteristics cannot be predicted in advance. The subsequent target-locked N2 and P3 potentials would instead reflect the involvement of either decision or response control processes that intervene to solve the interference arising after the target onset in the most demanding switch trials.

This EEG literature presents a more fragmented picture of the (domain-general) nature of task-switching processes than the fMRI studies mentioned above. This is due to the fact that relatively few ERP studies have purposely investigated whether different task-switching types rely on distinct or overlapping neural mechanisms (e.g., Hsieh and Wu, 2011). A common approach for ERP analysis has been, in fact, to pool the tasks in which participants had to perform a switch as long as the behavioral interaction between type of task and task requirements (repeat vs. switch) did not reach statistical significance (e.g., Goffaux et al., 2006; Nicholson et al., 2006; Karayanidis et al., 2009). This has usually been done to increase the signal-to-noise ratio of repeat and switch trials. Additionally, some of the previous ERP task-switching studies that explored the role played by the nature of the tasks used pursued different objectives with respect to the current rationale. For example, some researchers (e.g., Martin et al., 2011) have investigated the electrophysiological correlates of the so-called “asymmetrical switch cost”, which refers to a larger cost when participants switch to an easier task as compared to a more difficult one (e.g., Allport et al., 1994; Yeung and Monsell, 2003; Leleu et al., 2012). Accordingly, such studies were more interested in the influence that task dominance exerted on task-switching rather than the effects that distinct types of tasks could have on the neural markers of task-switching performance.

Other ERP studies have compared switching between stimulus-set and response-set dimensions (Rushworth et al., 2002, 2005; Hsieh and Wu, 2011), between single-shifts and dual-shifts (Tieges et al., 2007; West et al., 2009; Hsieh et al., 2014), and between the modality (visual vs. auditory) of the stimulus (Kieffaber and Hetrick, 2005). Collectively these studies provide mixed evidence on the domain-general nature of task-switching. For instance, whereas the switch positivity was found not to differ between stimulus-set and response-set switching (Hsieh and Wu, 2011), it was sensitive to the increased “task shift load” imposed by a dual-switch condition (Tieges et al., 2007). These divergent findings may be attributed to several factors, such as the use of different participants, task requirements, timing parameters, and complexity of task-set reconfiguration across the experiments that together hamper a direct comparison between them. It is also important to note that the definition of “task domain” changes markedly across studies, which makes it even more difficult to draw an unequivocal conclusion on what is meant by domain-general or domain-dependent. In the present context, and in agreement with our previous fMRI study (Vallesi et al., 2015), we followed a neural criterion to classify task domains. Specifically, participants had to switch either between two spatial tasks or between two verbal ones. Since processing of visuo-spatial information is usually more right-lateralized in the brain, whereas verbal processing mainly involves left-lateralized cognitive activities, this allowed us to investigate whether task-switching relies on common or distinct mechanisms independently of the strongly lateralized specific tasks to be performed.

To our knowledge, only two previous studies have addressed similar research questions. Miniussi et al. (2005) combined ERPs with a cued task-switching paradigm in which participants had to shift, on a trial-by-trial basis, between spatial and verbal (lexical) tasks that employed different stimulus materials. Conversely, Capizzi et al. (2015) recently devised a cued task-switching paradigm in which the same stimuli afforded spatial and verbal (semantic) categorization tasks. The results of the two studies differed in that, while in the former study the switch positivity was larger for the verbal as compared to the spatial task, in the latter it was found only for the spatial but not for the semantic task. Such differential outcomes thus call for further investigation of the possibly separate mechanisms mediating spatial and verbal task-switching.

Despite the above-mentioned differences between the two studies, which could be accounted for by several distinct experimental factors, a key aspect that should be taken into account regards the common use of a “between-domain” switch that could have also influenced task-switching processes. Specifically, it might be argued that because the participants of both studies had to switch between two different domains all the time (from verbal to spatial and vice versa), some carry-over interference effects would have come into play when moving attention from one domain to the other, and that this also had an influence on task-switching processes. To avoid this confounding feature, in the current study we kept the spatial and verbal domains among which participants had to switch separate across different blocks of trials. In such a way, we created a better-balanced setting to compare task-switching as a function of task domain.

Building upon our previous study (Vallesi et al., 2015), we predicted similar ERP modulations and time courses for task-switching in the spatial and verbal domains, which would further confirm the existence of domain-general task-switching processes. In particular, relative to a block fMRI design, the direct comparison of switch and repeat trials should help elucidate whether similar or distinct processes, concerning task-set reconfiguration and resolution of target interference, underlie both spatial and verbal task-switching. In order to fully investigate task-switching as a function of the two domains, we decided not to restrict the ERP analysis to a priori selected time windows and a small set of electrodes (e.g., Fz, Cz, and Pz), as is routinely done in the task-switching literature (e.g., Periáñez and Barceló, 2009; Androver-Roig and Barceló, 2010; Hsieh and Wu, 2011). Instead, we searched for differences in the entire spatio-temporal domains by using a mass univariate analysis approach (see Maris and Oostenveld, 2007), the details of which are presented below.

## Materials and Methods

### Participants

Fifty-four university students voluntarily took part in the experiment in exchange for a cash payment. All participants were native Italian speakers, with normal or corrected-to-normal visual acuity and color vision. They gave informed consent prior to their inclusion in the study, which was approved by the Bioethical Committee of the Azienda Ospedaliera di Padova and was conducted according to the guidelines of the Declaration of Helsinki. All participants reported no history of neurological or psychiatric disorders. Data from three participants were discarded due to poor ERP data quality (<30 artifact-free trials on at least one condition). We also rejected data from three additional participants because of low performance (accuracy level >2.5 SDs from the group mean on at least one condition). The data from the remaining 48 participants (mean age: 22.8 years, age range: 21–29 years, 14 men) were used for both behavioral and ERP analyses. All participants were right-handed according to the Edinburgh Handedness Inventory (Oldfield, 1971).

### Apparatus and Stimuli

Two Intel Core laptop computers with 17 inch screens were interconnected to run the experiment and to simultaneously record continuous electroencephalographic (EEG) activity. Stimulus presentation and data recording were controlled by E-prime 2 software (Schneider et al., 2002).

**FIGURE 1:**
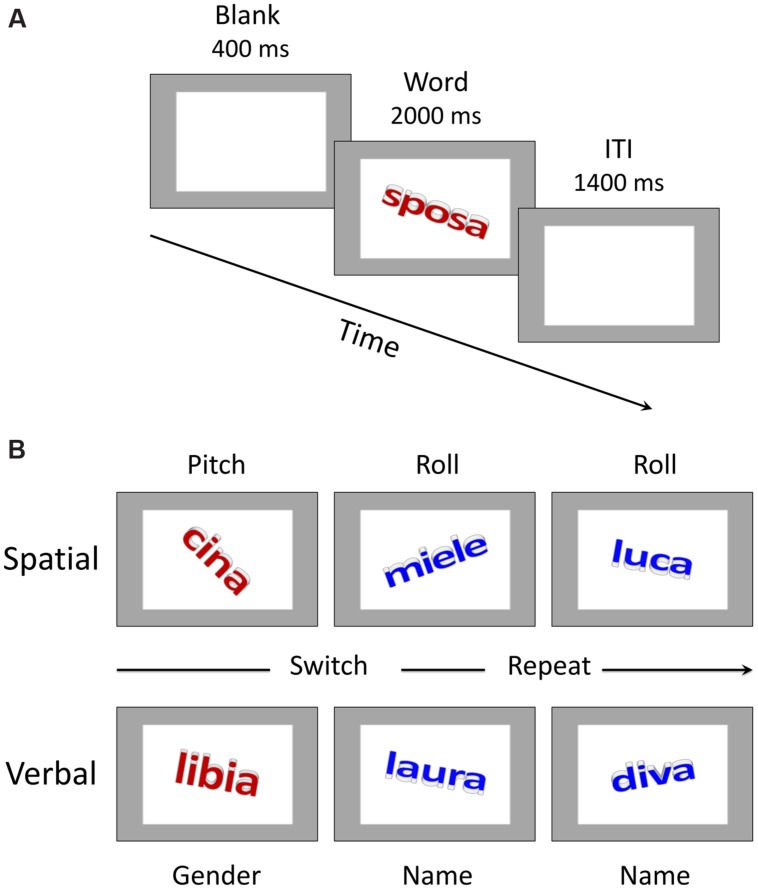
**Trial structure and sample stimuli.** **(A)** Schematic representation of events in a trial. ITI stands for inter-trial interval. “Sposa” is the Italian word for “bride”. **(B)** Examples of stimulus material for the spatial and verbal domains. The color of the word (red vs. blue) instructed participants about the specific task to be performed in the task-switching condition (please see the main text for further details). “Cina” is the Italian word for “China”, “miele” is the Italian word for “honey”, “luca” is the Italian version of the name “Luke”, “libia” is the Italian word for “Libya”.

A detailed description of the stimuli can be found in Vallesi et al. (2015) study. Briefly, there were 18 proper names, divided into 9 proper female names and 9 proper male names, and 18 common names, divided into 9 common female names and 9 common male names. The proper names consisted of personal names (e.g., “laura”) and names of states (e.g., “cina”, the Italian word for China), whereas the common names included generic terms denoting either non-living things (e.g., “miele”, the Italian word for honey) or people (e.g., “sposa”, the Italian word for bride). Both common and proper names were presented in lowercase letters in Calibri bold font and with a font size of 80, subtending on average a visual angle of 1.6° by 4.9°, viewed approximately 60 cm from the computer screen. All the words were created by adding a 3-D effect and a 3-D rotation, which allowed us to manipulate their physical spatial configuration. Specifically, each word could assume a clockwise or counterclockwise rotation (i.e., roll; for example, in **Figure 1B**, upper row, the words “cina” and “luca” show a clockwise rotation, whereas the word “miele” shows a counterclockwise rotation) and an upward or a downward rotation (i.e., pitch; in **Figure 1B**, upper row, the words “cina” and “miele” show a downward rotation, whereas the word “luca” shows an upward rotation). Each word could be written in one of four colors: red, blue, green or brown. The red and blue colors were associated with the task-switching condition (see below for further details), whereas the green and brown colors were used in the single-task condition. In the former case only, the colors signaled the task to be performed. In the latter case, the colors also changed from trial to trial in order to keep the perceptual characteristics of the stimuli similar across conditions, but participants were instructed not to pay attention to them.

### Procedure and Task

Participants were tested in a quiet and normally illuminated room. They were seated in front of the computer screen at a distance of approximately 60 cm. Both written and oral instructions were provided before the experimental session began. The task was the same as in Vallesi et al. (2015) with some minor differences concerning, for example, the duration of the inter-trial interval, which was longer in the fMRI study as compared to here. A trial started with the presentation of a 400 ms blank gray screen, which contained a gray frame lighter than the background color (see **Figure 1A**). After that time elapsed, the stimulus word, which was embedded inside the frame, was displayed for 2000 ms. Participants had to categorize the word according to the specific task instructions of each condition as outlined below. Following the period of 2000 ms, the next trial began after a 1400 ms inter-trial interval.

In the verbal single-task session, there were two subtasks that participants performed one at a time. The gender subtask required pressing the “f” key on the computer keyboard with the index finger of the left hand if the word was a female name and the “k” key with the index finger of the right hand if the word referred to a male name. In the name subtask, participants had to press the “f” key for a proper name and the “k” key for a common name. These category-response key assignments were counterbalanced across participants for each subtask condition. In the verbal task-switching condition, the color of the word instructed participants about the specific subtask they had to perform on any given trial. The blue color was associated with the name subtask in which participants had to decide whether the word referred to a proper name or to a common name. The red color instead signaled the gender subtask in which the decision regarded the female/male status of the word. The response keys were the same as those used for the verbal single-task conditions. As reported above, the word colors also changed randomly between brown and green in the single-task condition but in that case they had no bearing on the task.

The spatial-task session was similar to the verbal one and was implemented on exactly the same word stimuli. The spatial single-subtasks comprised a roll subtask in which participants had to classify the words according to their roll rotation (i.e., clockwise or counterclockwise) and a pitch subtask in which they had to respond to the pitch rotation (i.e., upward or downward). The keys for responses were the “f” key and the “k” key on the computer keyboard. The assignment of categories to response keys was counterbalanced across participants. In the task-switching condition, when the color was blue the task was to decide whether the word was rotated clockwise or counterclockwise, whereas when the color was red the task was to decide whether the word was rotated upward or downward (see **Figure 1B**).

Half of the participants started with the verbal-task session, whereas the other half started with the spatial-task one. Each session included two single-task blocks and four task-switching blocks each comprising 32 trials. Repeat and switch trials were presented in a pseudo-random order to guarantee roughly the same number of trials per block. Before the first and the third task-switching block were presented, participants performed a single-subtask according to the following order: single-task 1, task-switching 1, single-task 2, task-switching 2, 3 and 4. The first task-switching block was also preceded by 5 warm-up trials that allowed participants to refresh the corresponding stimulus-response mapping. The two single-task blocks were discarded from both behavioral and EEG analyses since the main focus of the current work was on the switch cost. Moreover, the verbal and spatial task-switching sessions were administered (in a counterbalanced order) with another type of executive function task requiring the monitoring of a spatial or a verbal target, which is not the object of the present study and whose results will be reported elsewhere.

Before the EEG recording, participants practiced both the verbal and spatial tasks. Each practice block comprised 10 trials. Participants received a feedback message (the Italian word for “wrong” displayed in red or the Italian word for “good” in blue) after their response on each trial for a duration of 1500 ms. In order to allow participants to fully process either the verbal or the spatial features of the words during the practice session, stimulus presentation was set to last until a button-press response was detected. However, if participants’ accuracy was below 80% after the block of practice trials, participants had to repeat the block until they reached this criterion. In these additional blocks, the stimulus duration was set to 2000 ms like in the proper experimental sessions. The experiment was automatically interrupted by the program if participants’ accuracy was still below 80% after 5 consecutive blocks of trials. All participants met this criterion and were proceeded to the subsequent EEG session.

### Electrophysiological Recording

Participants were seated in front of the computer monitor and were instructed to avoid eye blinks and movements during stimulus presentation. The EEG was recorded using BrainAmp amplifiers (Brain Products, Munich, Germany) from 64 Ag/AgCl electrodes that were mounted on an elastic cap (EASYCAP GmbH, Germany) according to the extended 10–20 system. Electrooculographic (EOG) activity was recorded with an electrode placed under the left eye and was also monitored through the scalp electrodes positioned in the proximity of both eyes. Impedances for each channel were measured and adjusted until they were kept below 10 kΩ before testing. All electrodes were referenced to FCz during the recording and were re-referenced off-line to the average of all electrodes. An electrode positioned at AFz served as the ground electrode. Raw data were band-pass filtered between 0.1 and 100 Hz and digitized at a sampling rate of 500 Hz.

### Data Analysis

#### Behavioral Data Analysis

Data from practice trials, the first trial of each block, trials with errors, and trials without responses were discarded from the analysis. Anticipated responses (RTs < 150 ms) were virtually absent. The mean RTs for correct responses were analyzed through a repeated-measures ANOVA with Domain (spatial, verbal) and Switching from the previous task (repeat, switch) as within-participants factors. Since accuracy data (percentage of correct responses) were not normally distributed (Shapiro–Wilk test, all *p*s < 0.08), we used a non-parametric Friedman’s ANOVA and follow-up Wilcoxon signed-rank tests to compare pairs of conditions.

#### Electrophysiological Data Analysis

Signal pre-processing was performed using BrainVision Analyzer 2.0 (Brain Products GmbH). Raw data were first filtered off-line with a 30-Hz low-pass filter (Butterworth zero phase, 48 dB/oct). An ocular correction algorithm based on independent component analysis (ICA) was performed on the continuous data to correct for eye movements and blink activity. Electrodes that were consistently bad according to the criteria described below during the entire recording were replaced through spherical spline interpolation (Perrin et al., 1989). Overall, only four electrodes (FP2, T7, AF8, and O2) were interpolated across four different participants. The data were then re-referenced to the average of all electrodes. They were finally segmented into epochs [-200, 1000 ms] with respect to the word onset. The period of 200 ms preceding the word onset was used to calculate the baseline.

Epochs were discarded if, on any channel, the absolute difference between two sampling points exceeded 30 μV/ms, if peak-to-peak deflections in a segment exceeded ±80 μV within intervals of 200 ms, if the amplitude exceeded a value of ±80 μV and if the activity was lower than 0.1 μV within intervals of 200 ms. Furthermore, each epoch was visually inspected and epochs containing any residual artifact were manually removed. A minimum of 30 trials per condition and participant was chosen as the criterion to ensure a sufficient signal-to-noise ratio. Only trials with correct behavioral responses were analyzed. In addition, practice trials and the first trial of each task-switching block were excluded from further analyses. Four separate grand average waveforms were constructed relative to our main experimental conditions: spatial-repeat, spatial-switch, verbal-repeat, and verbal-switch. The mean number of trials per participant (with ranges in parentheses) contributing to each grand average was as follows: 50 (34–59) for spatial-repeat, 55 (40–73) for spatial-switch, 56 for verbal-repeat (42–67) and 50 for verbal-switch (31–67).

Differences in the ERPs between the experimental conditions were tested for statistical significance through two-tailed non-parametric permutation tests based on the *t*_max_ statistic (Blair and Karniski, 1993). The analysis was performed using the Mass Univariate ERP toolbox written in Matlab with a family wise alpha level of 0.05 (Groppe et al., 2011a,b). The advantage of this statistical approach is that it avoids the a priori definition of time windows and/or scalp regions of interest, since the relevant univariate test comparing participants’ ERP amplitudes in different conditions (e.g., a paired *t*-test contrasting switch and repeat trials) is performed for each (channel, time)-pair. In our case, 32000 total comparisons were performed corresponding to the combination of the 64 channels used for the EEG recording and the 500 time points included between 0 and 1000 ms post-stimulus (i.e., the length of our segmentation). Each comparison was repeated 2500 times. Therefore, the most extreme *t*-value (i.e., the *t*_max_) in each of the 2500 permutations was used to estimate the *t*_max_ distribution of the null hypothesis against which to compare the 32000 observed *t* values. A particular benefit of using the *t*_max_ statistic is that it provides a strong control of the family wise error rate and thus a great degree of certainty that both the sign and the spatio-temporal localization of a given effect are reliable (Groppe et al., 2011a).

#### ERP-Behavior Correlation Analysis

Next, we performed two correlation analyses in order to investigate the association between behavioral and electrophysiological measures of participants’ task-switching performance. In the first analysis, we assessed the correlations between the mean RTs separately for switch and repeat trials and the corresponding ERP amplitudes for switch and repeat conditions. In the second analysis, we computed the correlation between the mean RT switch cost and the corresponding ERP switch effect, as given by the difference between switch and repeat trials in RTs and ERP amplitudes. For both analyses, we used the ERP amplitudes obtained in the mass univariate analysis. That is, the mean ERP amplitudes for both the switch and repeat conditions and for the ERP switch effects were calculated for the time points and across the channels showing the highest switch effect (i.e., the time points associated with the highest *t* values across those channels; see **Figure 3A**). In this sense, the correlational analyses were independent of our selection criteria, allowing us to avoid the circularity error (Kriegeskorte et al., 2010).

For both correlation analyses, we performed a Pearson’s correlation and conducted null hypothesis statistical significance testing by using a non-parametric percentile bootstrap test (2000 resamples; two-sided 95% confidence intervals (*B-CI*_95%_), corresponding to an alpha level of 0.05), which is more robust against heteroscedasticity compared with traditional *t*-tests (Pernet et al., 2013).

#### Source Estimation

Finally, we investigated the neuronal sources underlying the ERP components associated with task-switching performance. To this end, we performed cortical EEG source imaging on the individual ERPs in the switch and repeat conditions using Brainstorm, which is documented and freely available for download online under the GNU general public license (Tadel et al., 2011; http://neuroimage.usc.edu/brainstorm). A distributed source model consisting of 15002 elementary current dipoles was used to estimate the cortical current source distribution. These dipole sources were distributed at each node (i.e., vertex) of a tessellated cortical mesh template surface (brain model) derived from the FreeSurfer brain template (FSAverage) provided in Brainstorm (see Fischl et al., 1999). Dipole orientations were constrained to be normal to the surface of the cortex. The EEG forward modeling of volume currents was completed with a symmetric boundary element model generated with OpenMEEG using the adaptive integration method (Kybic et al., 2005; Gramfort et al., 2010). This volume conduction model of the head uses three realistic layers corresponding to the surface of the head (1922 vertices, relative scalp conductivity = 1), the outer skull (1922 vertices, relative skull conductivity = 0.0125), and the inner skull (1922 vertices, relative brain conductivity = 1). We estimated the current strength dynamics of the EEG cortical sources by using the depth-weighted minimum norm estimation approach as implemented in Brainstorm, with default parameter settings (Baillet et al., 2001). This technique has been shown to be robust to noise in recorded data and head model approximations with good spatial resolution. Also, the depth weighting used in this approach alleviates the natural bias of basic minimum norm estimation approaches toward superficial currents. A diagonal noise covariance matrix computed for each participant on pre-stimulus time points was used as an estimate of sensor variance. Next, the computed inverse estimator was used to transform each participant’s EEG time series in the switch and repeat conditions at each electrode into baseline-normalized (*z*-scored) dipole strengths. Finally, the *z*-map computed for each participant for the switch and repeat conditions was averaged over a time window of interest derived from the *t*_max_ permutation tests and compared using whole-brain two-tailed paired-sample *t*-tests. The results were then corrected for multiple comparisons using a cluster-based permutation test.

## Results

### Behavioral Results

#### Response Times

**FIGURE 2:**
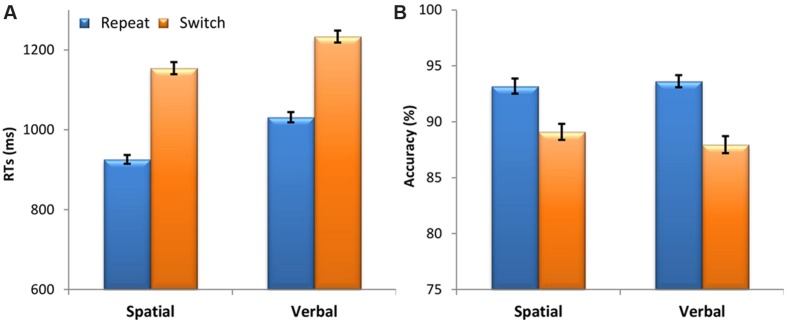
**Behavioral results.** **(A)** Mean response times (RTs) in milliseconds (ms) and **(B)** accuracy (percentage of correct responses) as a function of Domain (spatial, verbal) and Switching from the previous task (repeat, switch). Vertical bars represent the within-subjects standard errors of the mean (Morey, 2008).

Participants’ responses were longer for switch trials compared to repeat trials, demonstrated by a significant main effect of Switching [*F*(1,47) = 457.40, *p* = 7.3 × 10^-26^, $\eta_{p}^{2}$ = 0.91]. The main effect of Domain was also significant [*F*(1,47) = 23.52, *p* = 1.4 × 10^-5^, $\eta_{p}^{2}$ = 0.33] with shorter RTs for the spatial domain than for the verbal domain. The Domain by Switching interaction was not significant [*F*(1,47) = 1.87, *p* = 0.178, $\eta_{p}^{2}$ = 0.04], showing that the switch cost was comparable across the spatial and verbal cognitive domains (see **Figure 2A**).

#### Accuracy

A Friedman’s ANOVA showed that the level of accuracy significantly changed across domain and switching conditions [χ^2^(3) = 39.87, *p* = 1.1 × 10^-8^]. To follow up this finding, we used Wilcoxon signed-rank tests with a Bonferroni correction for multiple comparisons (α = 0.0125). These *post hoc* tests showed that accuracy was lower on switch trials compared to repeat trials for both the spatial (*z* = 4.02, *p* = 5.8 × 10^-5^) and verbal domains (*z* = 5.58, *p* = 2.4 × 10^-8^). There was no difference in the magnitude of the switch cost (switch – repeat) between the spatial and the verbal domain (*z* = 1.39, *p* = 0.165). Moreover, there was no difference between the spatial and verbal domains (*z* < 1) (see **Figure 2B**).

### Electrophysiological Results

We first tested for the statistical significance of the interaction between the Switching and Domain factors by performing a *t*_max_ permutation test contrasting the switch-repeat difference waves (i.e., the ERP switch effect) in the spatial and verbal cognitive domains. This analysis showed that not a single (channel, time)-data point reached the significance level (critical *t* value = ±4.88, *df* = 47, test-wise α level = 1.26 × 10^-5^), demonstrating that the ERP switch effect was comparable across the spatial and verbal cognitive domains. We therefore tested the statistical significance of the Switching and Domain main effects by performing *t*_max_ permutation tests contrasting, respectively, the ERPs for the switch and repeat conditions averaged across the two domains and the ERPs for the spatial and verbal domains averaged across the two switching conditions. Since the main effect of domain was beyond the scope of the present work, the results concerning this effect are not reported.

**FIGURE 3:**
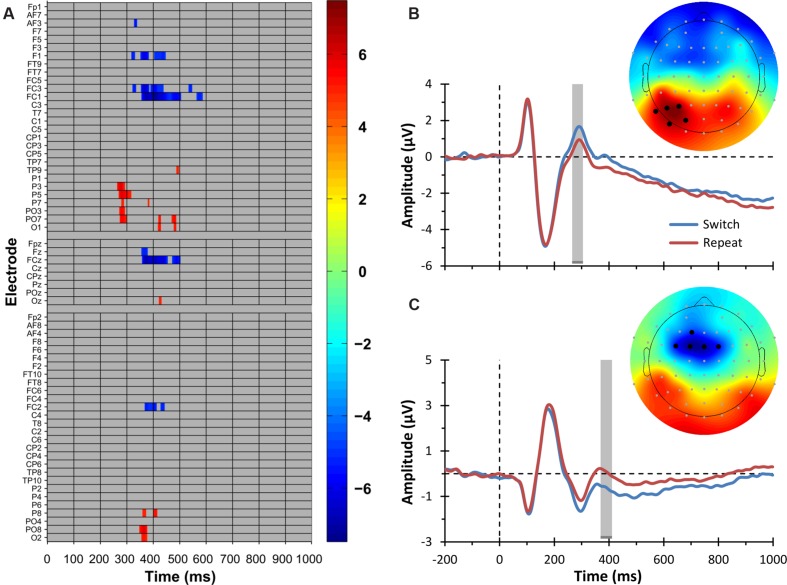
**Electrophysiological results: switching main effect.** **(A)** Raster diagram showing significant differences between ERPs elicited by switch and repeat trials (i.e., the ERP switch effect) according to the *t*_max_ permutation test. Rectangles in warm and cold colors indicate electrodes/time points in which the ERPs to switch trials are more positive or negative, respectively. The colorbar on the right indicates *t* values. Gray rectangles indicate electrodes/time points at which no significant differences were found. Note that the electrodes are organized along the y-axis somewhat topographically (Groppe et al., 2011a). Electrodes on the left side of the head are grouped on the top part of the diagram, those on the right side on the bottom, and midline electrodes are shown in the middle. Within those three groupings, the y-axis top-to-bottom corresponds to scalp anterior-to-posterior. **(B)** The trace plot depicts the grand average ERPs to switch and repeat trials pooled over the electrodes showing the parieto-occipital ERP switch effect, namely, P3, P5, P7, PO3, and PO7. These electrodes are indicated as black circles in the topographical map on the right. The topographical map shows the *t* values for the parieto-occipital ERP switch effect in a 30-ms time window centered on the time point at which the ERP switch effect was maximal. This time window is indicated by the gray shaded region in the ERP plot. The color scale is the same as that of the raster diagram. **(C)** The trace plot depicts the grand average ERPs to switch and repeat trials pooled over the electrodes showing the frontal ERP switch effect, namely, F1, FC3, FC1, FCz, and FC2. These electrodes are indicated as black circles in the topographical map on the right. The topographical map shows the *t* values for the frontal ERP switch effect in a 30-ms time window centered on the time point at which the ERP switch effect was maximal. This time window is indicated by the gray shaded region in the ERP plot. The color scale is the same as that of the raster diagram. Note that the time windows indicated by the gray shaded regions in **(B,C)** are the same as those used in the source estimation analysis (see **Figure 5**).

The mass univariate analysis of the Switching main effect revealed several significant differences between switch and repeat ERPs (critical *t* value = ±4.94, *df* = 47, test-wise *α* < 1.03 × 10^-5^). In particular, the *t*_max_ permutation test showed two principal electrophysiological modulations. As portrayed in **Figure 3A** (warm color), the first modulation concerns significantly more positive ERP amplitudes for the switch compared to the repeat condition during a time window ranging from 265 to 310 ms over left parieto-occipital electrodes (P3, P5, P7, PO3, PO7). **Figure 3B** shows the corresponding topographic map and the grand average ERP for this result. The second modulation, represented in **Figures 3A** (cold color) and **3C**, concerns a slow-wave ERP developing during a later time window (350–450 ms). This deflection was characterized by more negative ERP amplitudes for the switch compared to the repeat condition over mid-left fronto-central electrodes (FC1, FC3, FCz, F1) and over FC2, which remained significant up to 500 ms only over F1 and FCz electrodes.^1^ Correspondingly, three opposite short-lived effects, with a more positive ERP amplitude for the switch compared to the repeat condition, were observed (i) at the 350–376 ms time points over right posterior electrodes (O2, PO8), (ii) around 425 ms over mid-left posterior electrodes (Oz, O1, PO7), and (iii) around 480 ms over left posterior electrodes (O1, PO7).

**FIGURE 4:**
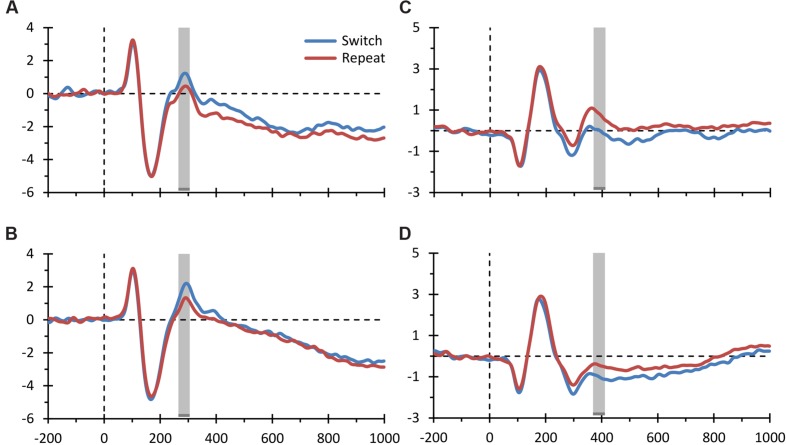
**Event-related potential (ERP) switch effects for the spatial and verbal domains.** The figure displays the grand average ERPs elicited by switch and repeat trials plotted separately for the spatial and verbal domains for both the parieto-occipital ERP switch effect (**A,B** depict the spatial and verbal domains, respectively) and the frontal ERP switch effect (**C,D** depict the spatial and verbal domains, respectively) shown in **Figures 3B,C**. The conventions are the same as those of **Figure 3**.

**Figure 4** displays the grand average ERPs for the two main electrophysiological switch-related effects described above, separately for the verbal and spatial domains. These graphs further illustrate that the two domains showed comparable electrophysiological modulations. In order to statistically verify this visual inspection of the data, we calculated an effect size estimate for both the parieto-occipital and the frontal ERP switch effects. That is, we computed the maximum value of Cohen’s d in the 30 ms-wide spatio-temporal windows used in our analysis [80 (channel, time)-data points: five channels ^∗^ 16 samples] for (i) the switch main effect, (ii) the switch effect for the verbal domain, and (iii) the switch effect for the spatial domain. For the parieto-occipital ERP switch effect, we found a large effect size for the main effect (*d* = 0.859) and large effect sizes for both the spatial and verbal domains (respectively, *d* = 0.582 and 0.762). The same pattern was observed for the frontal ERP switch effect, with a large effect size for the main effect (*d* = -1.08) and large effect sizes for both the spatial and verbal domains (respectively, *d* = -0.759 and -0.663). These secondary analyses thus corroborate the main finding described above that switch-related ERP components were similarly modulated in the verbal and spatial domains.

### ERP-Behavior Correlation

The correlation analyses revealed that the earlier parieto-occipital ERP switch effect did not significantly correlate with the RT switch cost (*r* = -0.020, *B-CI_95%_* = -0.261 to 0.218, *p* = 0.852). Conversely, the later frontal ERP switch effect was significantly correlated with the RT switch cost (*r* = 0.254, *B-CI_95%_* = 0.024 to 0.469, *p* = 0.039). That is, participants who showed larger switch–repeat differences in the later frontal ERP component (i.e., those who showed more negative frontal ERPs in the switch condition compared to the repeat one) showed a smaller RT switch cost, indicating that they were more able to flexibly switch between tasks.

With regard to the correlations for the switch and repeat conditions taken separately, the analyses revealed that both the parieto-occipital and frontal ERP amplitudes for the switching condition were significantly correlated with the mean RT in the same condition, though a positive correlation was seen with the parieto-occipital ERP and a negative correlation with the frontal ERP (respectively, *r* = 0.404, *B-CI_95%_* = 0.163 to 0.580, *p* = 0.003; and *r* = -0.328, *B-CI_95%_* = -0.571 to -0.013, *p* = 0.041). The same pattern was observed for the repeat condition (respectively, *r* = 0.328, *B-CI_95%_* = 0.050 to 0.551, *p* = 0.036; and *r* = -0.336, *B-CI_95%_* = -0.545 to -0.054, *p* = 0.012).

### Source Estimation Results

**FIGURE 5:**
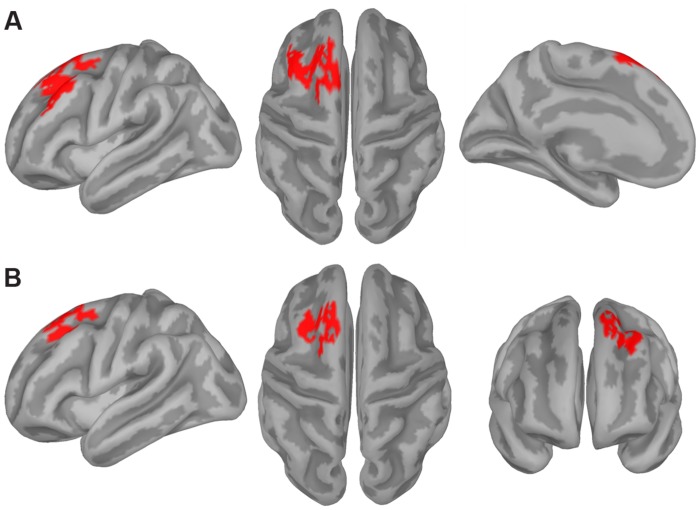
**Source estimation results.** The figure displays the significance mask representing the cluster of cortical sources showing a statistically significant switch effect (switch > repeat) for the frontal **(A)** and posterior **(B)** ERP switch effect as revealed by the cluster-based permutation test. This test compared the *z*-maps representing the estimated activity of the cortical sources in the repeat and switch conditions in the 30-ms time window corresponding to the ERP switch effects revealed by the *t*_max_ permutation test (see gray shaded area in **Figures 3B,C**, respectively).

First, we computed the individual functional source activation maps (i.e., the *z*-maps) underlying the frontal ERP switch effect since the correlation analysis showed it to be significantly correlated with the RT switch cost. To this end, we averaged the individual *z*-maps for the switch and repeat conditions over a 30 ms time window^2^ centered on the time point at which the frontal ERP switch effect was maximal (i.e., the same time point used in the ERP-behavior correlational analysis). The cluster-based permutation analysis contrasting these *z*-maps revealed a significant difference between the switch and repeat conditions in a cluster of adjacent dipoles distributed over the left prefrontal cortex (PFC) including, in particular, the middle frontal gyrus (MFG), the superior frontal sulcus (SFS), and the superior frontal gyrus (SFG), which contained the pre-supplementary motor area (pre-SMA) on its medial aspect (**Figure 5A**).

We also performed the same analysis on a 30-ms time window centered on the time point at which the parieto-occipital ERP switch effect was maximal (i.e., again, the same time point used in the ERP-behavior correlational analysis). The cluster-based permutation analysis contrasting the *z*-maps for the switch and repeat conditions revealed a significant difference between the two conditions in a cluster of adjacent dipoles distributed over the left PFC including the dorsal-most part of the MFG, the SFS, and the SFG (**Figure 5B**).

Although the two clusters shown in **Figure 5** look quite similar, as both were distributed over the left dorsolateral PFC, they differed in the following aspects. The clusters related to the parieto-occipital and frontal ERP switch effects consisted of 117 and 188 vertices, respectively, and the spatial overlap between them involved 95 vertices, corresponding to 50.5% of those composing the frontal ERP switch effect. In particular, the frontal cluster involved the lateral aspect of the left MFG and the medial aspect of the left SFG, which were not included in the parieto-occipital ERP switch effect cluster.

It should be also noted that the topographies of the switch-related cortical activations for the two ERP switch effects differed even more when comparing the two distributions over the entire cortical surface (i.e., across the 15002 current dipoles). For instance, the parieto-occipital ERP switch effect was specifically related to switch-related activations in clusters of dipoles distributed over bilateral posterior parietal cortex and paracentral gyri, as well as over the right inferior frontal gyrus, whereas the frontal ERP switch effect was specifically related to stronger switch-related activations in clusters of dipoles distributed over the left inferior frontal gyrus. These effects, however, were not reported as they did not survive correction for multiple comparisons.

## Discussion

The current study investigated the electrophysiological correlates of task-switching in the context of spatial and verbal cognitive domains and sought to identify the underlying neural sources. As in our previous neuroimaging work (Vallesi et al., 2015), the same stimulus material was administered for both the spatial and verbal domains to assure identical physical stimulation across the two types of task-switching. Domain was manipulated in different blocks of trials in order to avoid any possible carry-over interference effect related to the movement of attention from one domain to the other (see Capizzi et al., 2015).

In our task-switching paradigm, the task cue was provided simultaneously with the target as the color of the stimulus word indicated the specific spatial or verbal task to be implemented on any given trial (e.g., Friedman et al., 2008; Gajewski et al., 2010). It follows that participants were not afforded anticipatory preparation before the target onset as the word color was the only task cue presented. At the behavioral level, this manipulation resulted in a large switch cost in both domains (228 ms in the spatial domain and 203 ms in the verbal domain), though overall RTs were longer in the verbal domain (see Miniussi et al., 2005; Capizzi et al., 2015). Importantly, the magnitude of the switch cost did not differ between the spatial and verbal domains as demonstrated by the lack of a significant Domain by Switching interaction. Regarding the accuracy data, there was no difference between the two domains suggesting that, although participants took longer to perform the verbal task, this did not influence their accuracy level.

At the electrophysiological level, the spatially- and time-uninformed mass univariate approach that was applied to the ERP data highlighted three critical results. First, the *t*_max_ permutation test performed to explore the Switching by Domain interaction mirrored the RT data by showing no differences between the spatial and verbal domains in the modulation of switch and repeat ERPs. Second, the *t*_max_ permutation test contrasting the ERPs elicited by switch and repeat trials showed that switch trials were associated with a more positive amplitude compared to repeat trials. The positive ERP differential deflection was the first component to appear. It developed in the time window ranging from 265 to 310 ms and was distributed over left parieto-occipital electrodes. The third result concerned a subsequent slow-wave modulation (350–450 ms) occurring mostly over mid-left fronto-central electrodes, which was characterized by a more negative ERP amplitude for the switch compared to the repeat condition.

The finding that the first ERP deflection was relatively more positive for switch compared to repeat trials is reminiscent of the well-known switch positivity modulation reported in cued task-switching paradigms. Previous ERP studies have documented a larger parietal positivity developing during the cue-target interval in response to a switch-cue compared to a repeat-cue (e.g., Karayanidis et al., 2010). There is agreement that such a switch positivity is composed by multiple sub-processes marking distinct aspects of task-set reconfiguration. For instance, Nicholson et al. (2006; see also Karayanidis et al., 2009, for a follow-up study) showed that some reconfiguration processes could occur either in advance of or after target onset depending on the amount of information available during the preparation interval. This was shown through a paradigm that employed three different types of tasks and three types of cues, namely, a “repeat” cue indicating a task repetition, a “switch-to” cue indicating the specific task to switch to on the next trial, and a “switch-away” cue indicating simply a switch away from the previous task without specifying which of the two remaining tasks should be performed on the next trial. On this ground, our findings of a larger positivity after a switch (cue) color compared to a repeat (cue) color fits well with the idea that switching to a different task would firstly necessitate the reconfiguration of the task-set that was used on the previous trial. Importantly, our study also shows that such processes are domain-general, at least when switching requirements are manipulated in a within-domain switch design (cf., Capizzi et al., 2015).

It is, however, worth noting that some results regarding the task-set reconfiguration observed here diverged from previous cued task-switching studies that used a preparation interval. Specifically, early research analyzing the switch positivity elicited during the cue-target interval reported that RTs on switch trials were negatively correlated with both the cue-locked positivity amplitude for switch trials and the amplitude of the switch-repeat differential positivity, whereas no correlation was found between RTs on repeat trials and the positivity amplitude for repeat trials (Karayanidis et al., 2011). That is, participants who were faster on switch trials and had a smaller RT switch cost also displayed a larger positivity for switch trials as well as a larger switch-repeat differential positivity. These results were taken as evidence that the switch positivity reflected the involvement of an advance reconfiguration process that was specifically elicited by switch trials and whose successful engagement was associated with a reduction of the RT switch cost. Unlike these findings, in the context of our overlapping cue-target design, the parieto-occipital ERP switch effect (i.e., the switch-repeat difference) did not correlate with the RT switch cost. Moreover, both RT switch and repeat trials were positively, instead of negatively, correlated with the positivity amplitude of switch and repeat conditions, respectively. Thus, as shown by the correlation analyses, the switch positivity found here may be better interpreted as reflecting the endogenous, time-consuming process related to task-set reconfiguration. Indeed, it is plausible to speculate that under the demands of our extremely difficult task-switching paradigm, which did not afford anticipatory preparation, the more effortful process was the activation of the task-set, as shown by the positive correlation between RTs and the amplitude of the positivity on switch trials. An analogous positive correlation was found for the repeat condition, which may have also required the involvement of some reconfiguration processes to maintain the correct task-set properties.

In line with the above-mentioned idea that switching between spatial or verbal tasks required an initial endogenous control process, the cortical source reconstruction of the switch positivity identified an increased cortical activity for switch trials in left dorsolateral prefrontal areas (see **Figure 5B**), which have been shown to be involved in cognitive control (e.g., Cole and Schneider, 2007) and in task-switching abilities (e.g., Braver et al., 2003; Ruge et al., 2005). The left lateralization of such frontal areas also replicates our previous fMRI study (Vallesi et al., 2015), although the left frontal sources found here (see also the source results for the frontal ERP switch effect) were more dorsally located than the peak voxel shown by the fMRI analysis (i.e., left inferior prefrontal cortex). The difference between the two studies may be due to the fact that Vallesi et al. (2015) used a block design in which they contrasted task-switching vs. single-task blocks, which may have also influenced the specific localization of brain activity. Moreover, it is important to keep in mind that functional localization of ERPs presents some limitations that need to be taken into account when drawing a parallel between the outcomes of the two methodologies. With the proper caution, however, it is worth emphasizing that the involvement of left prefrontal areas in task-switching seems to be domain-general as it was found in the context of both source reconstruction of transient ERP activity and in fMRI data.

The second significant ERP component (350–450 ms) that differentiated switch and repeat trials was a negative deflection occurring mostly over mid-left fronto-central scalp regions that was larger for the switch compared to the repeat condition. The fact that switch trials showed a relative reduction in positivity compared to repeat trials is in line with previous target-locked ERP findings obtained in a typical non-overlapping cue-target interval paradigm. However, while in these former studies such a reduced positivity usually manifested itself as a smaller centro-parietal P3 amplitude for switch relative to repeat trials (e.g., Kieffaber and Hetrick, 2005; Gajewski and Falkenstein, 2011; Hsieh and Wu, 2011), here instead it was expressed solely as an increased fronto-central negativity for the switch condition. The lack of a cue-target interval may help explain why we did not find the typical morphology of the target-locked P3 potential (see also Friedman et al., 2008). The frontal scalp distribution of our switch effect suggests that it could belong to the family of N2-like potential waveforms, which are usually elicited in experimental paradigms that require a great deal of executive control to overcome interference that arises at the level of decision and response stage processes, such as in the Eriksen Flanker task (see Folstein and van Petten, 2008). Supporting this, a larger fronto-central N2 for the more demanding switch trials compared to the less demanding repeat ones has also been reported in previous task-switching paradigms (see Karayanidis and Jamadar, 2014). Therefore, it is likely that in the present context in which participants had no opportunity for anticipatory preparation, a successful switch depended on the involvement of strong control mechanisms in charge of resolving interference and associating the imperative rule with the exact response mapping. This hypothesis is bolstered by two observations. First, the frontal ERP switch effect was negatively correlated with the RT switch cost, such that participants showing a larger switch-repeat difference in the frontal ERP component also showed a smaller RT switch cost. This means that they were better able to overcome the interference related to task-switching requirements. Second, the prefrontal areas found in the source reconstruction of the frontal switch effect, such as the pre-SMA (see **Figure 5A**), are in line with traditional neural regions involved in overcoming task-set interference and in implementing the correct stimulus-response mapping (e.g., Dove et al., 2000; Rushworth et al., 2002; Yeung et al., 2006; Vallesi et al., 2015). Once again, the left lateralization of this cortical source lends support to the idea that a left-lateralized set of brain areas could mediate the ability to flexibly switch between tasks. This finding thus adds to previous block fMRI studies by directly contrasting the transient activity elicited by switch relative to repeat trials across distinct domains. Accordingly, a general conclusion from our work would be that the left lateralization pattern usually associated with task-switching is process-based and does not depend on the domain of the tasks employed in switching (see Ambrosini and Vallesi, 2016, for compatible results with a spectral EEG analysis). However, future research is needed to extend this conclusion to other domains manipulating different stimulus materials. That is, since the spatial and verbal tasks used here both administered exactly the same visual stimuli, one could argue that a complementary test of the domain-general nature of task-switching would be to use more dissimilar stimulus categories that draw on distinct neural areas (for example faces vs. objects usually associated with more right and left hemispheric dominance, respectively; e.g., Orban and Caruana, 2014; Frässle et al., 2016). Finding that the ERP correlates of task-switching would not differ for both similar and different types of stimulus materials would provide further evidence for its plausible domain-general nature.

As a final remark, it should be noted that another way of investigating the domain-general nature of task-switching would be to separate cue and target stimuli by a long preparation interval. Indeed, it makes sense to suggest that presenting cue and target within the same time frame does not allow one to completely disentangle the processes triggered by the cue from those related to target onset. Despite the fact that the ERP results obtained in the context of our overlapping cue-target design showed two temporally distinct switch-specific brain potentials, it would be informative to compare these outcomes with those typically obtained within a long cue-target interval.

## Conclusion

The ERP findings reported here are coherent with previous research showing that task-switching is mediated by multiple processes related to task-set reconfiguration and resolution of interference. Importantly, our data suggest that both kinds of processes are subserved by left-lateralized brain areas that operate regardless of the specific cognitive domain in which switching occurs.

## Author Contributions

MC drafted the manuscript and was involved in all subsequent revisions. She was also involved in the conception of the work, creation of stimuli and materials, data collection, and data analysis. EA drafted the manuscript, was involved in all subsequent revisions, and performed statistical analyses. SA was involved in the conception of the work, creation of stimuli and materials, and data collection. She also provided additional revisions to the manuscript. IM performed analysis of data and provided additional revisions to the manuscript. AV was involved in the conception and design of the work and provided ongoing contributions and feedback throughout the experimental process. He also provided additional revisions to the manuscript. All the authors have approved the final version of the manuscript, and agree to be accountable for all aspects of the work.

## Conflict of Interest Statement

The authors declare that the research was conducted in the absence of any commercial or financial relationships that could be construed as a potential conflict of interest.

## Footnotes

1 One could argue that our findings of positive posterior and negative frontal modulations could be due to the average reference we used, which can produce reversed fronto-parietal potentials reflecting the same underlying cognitive process. In order to explore this possibility and to better compare our task-switching ERP study with previous research that has mainly used a linked mastoids re-reference, we also computed and plotted the ERP traces with an average mastoids re-reference (e.g., Picton et al., 2000). As depicted in the Supplementary Figure S1, the resulting ERPs still showed an early parieto-occipital ERP switch effect followed by a frontal ERP switch effect with a mirrored pattern of results (thus ruling out the possibility that our findings can be accounted for just by the average reference we used).

2 Here we used a time window instead of a single time point to increase the statistical power. The length of the time window was chosen to include all the time points for which at least two electrodes showed a significant ERP switch effect in the mass univariate analysis.
