# Supplementary material for: Electrophysiological Evidence for Domain-General Processes in Task-Switching
Source: Front Hum Neurosci. 2016 Mar 21;10:124. doi: 10.3389/fnhum.2016.00124 (PMC4800168; doi:10.3389/fnhum.2016.00124)
Supplement: Supplementary file 1 [file Image_1.PDF]

Supplementary Material

Electrophysiological Evidence for Domain-general Processes in Task-switching

Mariagrazia Capizzi*, Ettore Ambrosini*, Sandra Arbula, Ilaria Mazzonetto, Antonino Vallesi

*** Correspondence:** Mariagrazia Capizzi: mariagrazia.capizzi@unipd.it Ettore Ambrosini: ettore.ambrosini@unipd.it

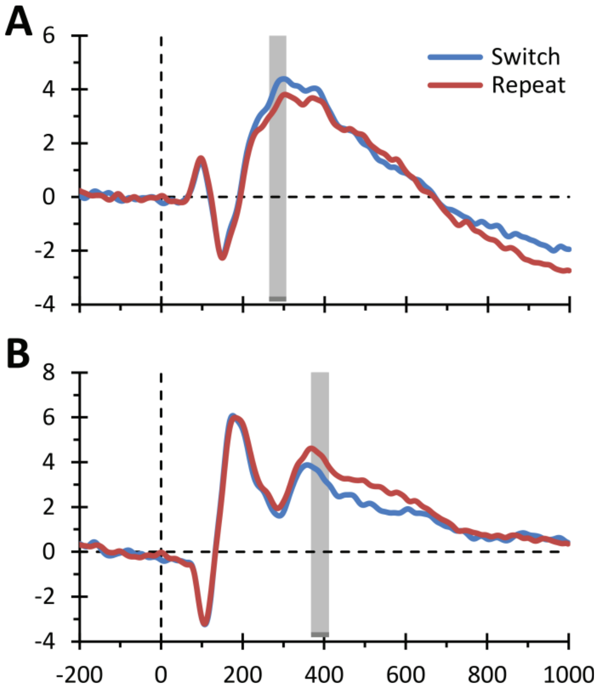

Supplementary Figure S1. ERP switch effects re-referenced to average mastoids. The trace plots depict the grand average ERPs elicited by switch and repeat trials for the parieto-occipital (A) and frontal (B) ERP switch effect shown in Figure 3B and 3C, respectively, after re-referencing to average mastoids. To facilitate comparison, the gray shaded regions are the same as those used in Figure 3.
